# Ruminal microbe of biohydrogenation of trans-vaccenic acid to stearic acid in vitro

**DOI:** 10.1186/1756-0500-5-97

**Published:** 2012-02-15

**Authors:** Dan Li, Jia Qi Wang, Deng Pan Bu

**Affiliations:** 1 State Key Laboratory of Animal Nutrition, Institute of Animal Science, Chinese Academy of Agricultural Sciences, Beijing, 00193, P. R. China; 2 Guansu Agricultural Universtity, Lanzhou, Gansu, 730070, China

**Keywords:** Ruminal microbe, Biohydrogenation, Trans-vaccenic acid

## Abstract

**Background:**

Optimization of the unsaturated fatty acid composition of ruminant milk and meat is desirable. Alteration of the milk and fatty acid profile was previously attempted by the management of ruminal microbial biohydrogenation. The aim of this study was to identify the group of ruminal trans-vaccenic acid (trans-11 C18:1, t-VA) hydrogenating bacteria by combining enrichment studies in vitro.

**Methods:**

The enrichment culture growing on t-VA was obtained by successive transfers in medium containing t-VA. Fatty acids were detected by gas chromatograph and changes in the microbial composition during enrichment were analyzed by denaturing gradient gel electrophoresis (DGGE). Prominent DGGE bands of the enrichment cultures were identified by 16S rRNA gene sequencing.

**Results:**

The growth of ruminal t-VA hydrogenating bacteria was monitored through the process of culture transfer according to the accumulation of stearic acid (C18:0, SA) and ratio of the substrate (t-VA) transformed to the product (SA). A significant part of the retrieved 16S rRNA gene sequences was most similar to those of uncultured bacteria. Bacteria corresponding to predominant DGGE bands in t-VA enrichment cultures clustered with t-VA biohydrogenated bacteria within Group B.

**Conclusions:**

This study provides more insight into the pathway of biohydrogenation. It also may be important to control the production of t-VA, which has metabolic and physiological benefits, through management of ruminal biohydrogenation bacterium.

## Background

Unsaturated fatty acids are present at low concentrations in meat and milk. As the consumption of dairy products and ruminant meats is often associated with an increased incidence of coronary heart disease [1] (Menotti and Kromhout et al., 1999), the transformation of unsaturated fatty acids to saturated fatty acids, or biohydrogenation, in ruminants represents a major human health issue. The biohydrogenation process has long been known to occur in the rumen as the result of microbial metabolic activity [2] (Jenkins and Wallace et al., 2008).

Microbial biohydrogenation is the process of converting unsaturated fatty acids to more saturated end products by gut microbes. Biohydrogenation is a unique biological process prevalent in the microbial ecosystem found within the rumen. In cows fed a typical forage diet, the major biohydrogenation intermediate present in ruminal contents is *trans*-vaccenic acid (*trans*-11 C18:1, t-VA) [3] (Bickerstaffe and Noakes et al., 1972). t-VA serves as a precursor for the synthesis of saturated fatty acid in the rumen and of conjugated linoleic acid (CLA) at the tissue level. t-VA is reduced in the rumen to form stearic acid or is desaturated by Δ^9^-desaturase in the mammary tissue to produce cis-9, trans-11 C18:2, which is an abundant CLA isomer in meat and milk [4] (Griinari and Bauman, 1999). Thus, if ruminal biohydrogenation of unsaturated fatty acids, especially t-VA, can be controlled, it may be possible to improve the healthfulness of ruminant meats and milk by increasing their unsaturated fatty acid composition in general and CLA in particular [5,6] (Griinari and Corl et al., 2000; Scollan and Choi et al., 2001).

Kemp and Lander [7] (1984) divided ruminal bacteria into two groups based on the reactions and end products of biohydrogenation. Group A bacteria were able to hydrogenate linoleic acid and linolenic acid, with t-VA being their major end product. Group B bacteria utilized t-VA as one of the main substrates with SA being the end product.

The objectives of the current study were to identify the group composition of t-VA hydrogenating bacteria when t-VA was the exclusive substrate in vitro, and characterize bacterium diversity by denaturing gradient gel electrophoresis (DGGE).

## Results

### Metabolism of trans-vaccenic acid to stearic acid

**Figure 1:**
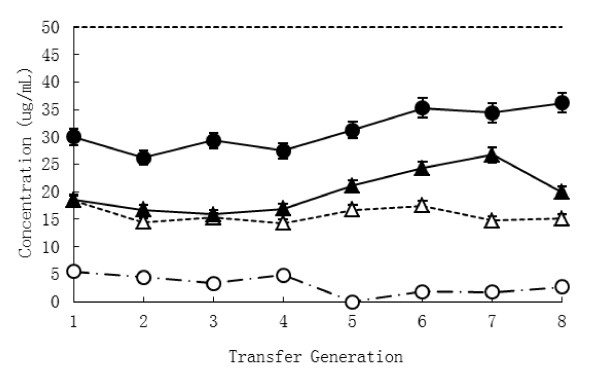
**Accumulation of SA and concentration of t-VA during successive transfers**. △concentration of SA in culture without t-VA (control group, CK), ○concentration of t-VA in culture without t-VA (control group, CK), ▲concentration of SA in culture with t-VA (treatment group, tVA+), ●concentration of t-VA in culture with t-VA (treatment group, tVA+); 1 to 8 represent transfer generation.

**Figure 2:**
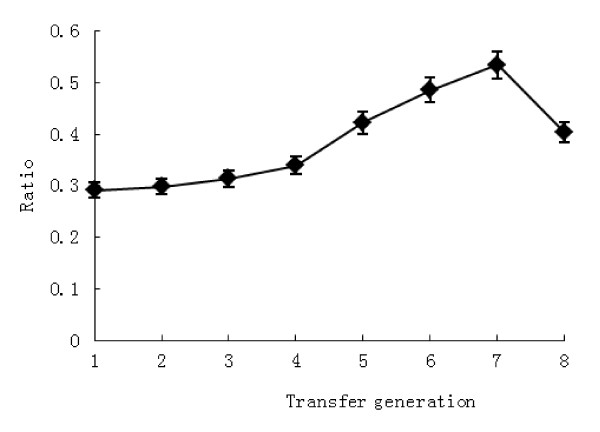
**Ratio of t-VA conversion to SA by generation culture in the t-VA treatment group 1 to 8 represent transfer generation**.

t-VA enrichment cultures were obtained through numerous transfers (n = 8) into fresh medium containing t-VA (50 μg mL^-1^), whereas cultures without t-VA served as the control group. Incubation was performed at 39°C, and the accumulation of SA as the main metabolism product in the culture and concentration of t-VA as substrate was collected at each transfers (Figure 1). In the treatment group, the concentration of SA before the third transfer decreased slightly. After that, the accumulation of SA increased gradually at successive transfer times compared to the control group. The concentration of SA was highest in the treatment group (26.69 ± 0.31; mean ± SEM), significantly higher than the control group (14.74 ± 0.27; mean ± SEM) at the seventh transfer (*P *= 0.05). Meanwhile the concentration of t-VA in the treatment group increased slightly at subsequent transfers, though there was no obvious difference between transfers. Rumen fluid, a culture medium component, contained limited SA. In order to overcome the influence of SA contained in culture medium, the ratio of t-VA conversion to SA was counted, which eliminated the effect of the medium. The result showed that the ratio of conversion obviously increased over time (Figure 2). There was the highest at the seventh transfer compared to other transfers (*P *= 0.03). The analysis showed that interaction between supplementation of t-VA and transfer times were significantly different (*P *< 0.05). The trend of SA accumulation (Figure 1) was the same as the ratio of t-VA conversion to SA (Figure 2).

### DGGE analysis

**Figure 3:**
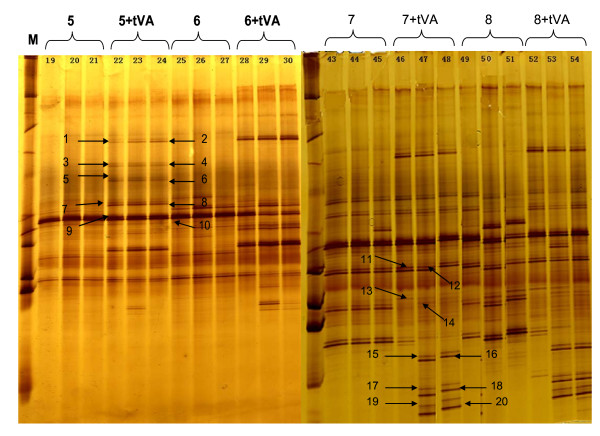
**Bacterial DGGE patterns of the t-VA enrichment by successive transfers in anaerobic media M, marker; x(+tVA), successive transfer, where x represents the number of transfers**. Closest relative species of the selected clones, determined by an NCBI BLAST search.

Microbial diversity and shifts in bacterial communities during the selective enrichment of t-VA were monitored by DGGE fingerprinting of PCR-amplified 16S rRNA gene fragments (Figure 3). In the enrichment series, less complex DGGE patterns were observed after each transfer with a reduction in the number of predominant bands indicating a rapid reduction in the bacterial richness. DGGE banding patterns from the fifth to eighth transfers are shown in Figure 3. The DGGE profile from the first to second transfer did not showed any obvious difference between control and treatment groups (data not shown). For the t-VA enrichment series, the 16 bands in the bacterial richness compared to the control was already verified, and 4 bands (bands 9, 10, 11, 12) appeared to exist in both control and treatment groups during the successive enrichments (Figure 3).

**Figure 4:**
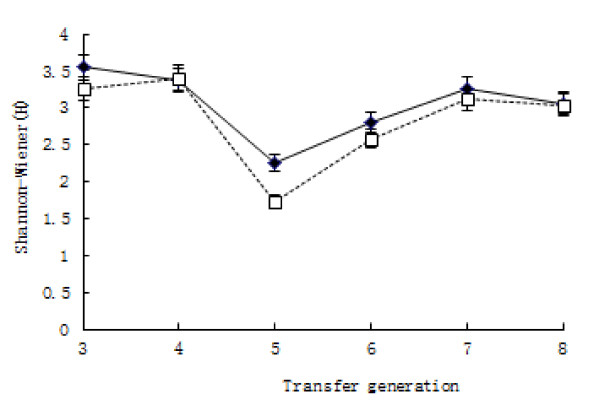
**Shannon diversity indices (*H*) during successive transfers ♦culture with t-VA (tVA+), ☐culture without t-VA (CK); 3 to 8 represent transfer generation**.

Shannon diversity indices (H) calculated based on DGGE patterns obtained for each of the enrichment samples evidenced a decrease in bacterial diversity over time, as expected (Figure 4). Diversity indices between t-VA culture and control culture were not different at each transfer point (*P *= 0.063).

**Figure 5:**
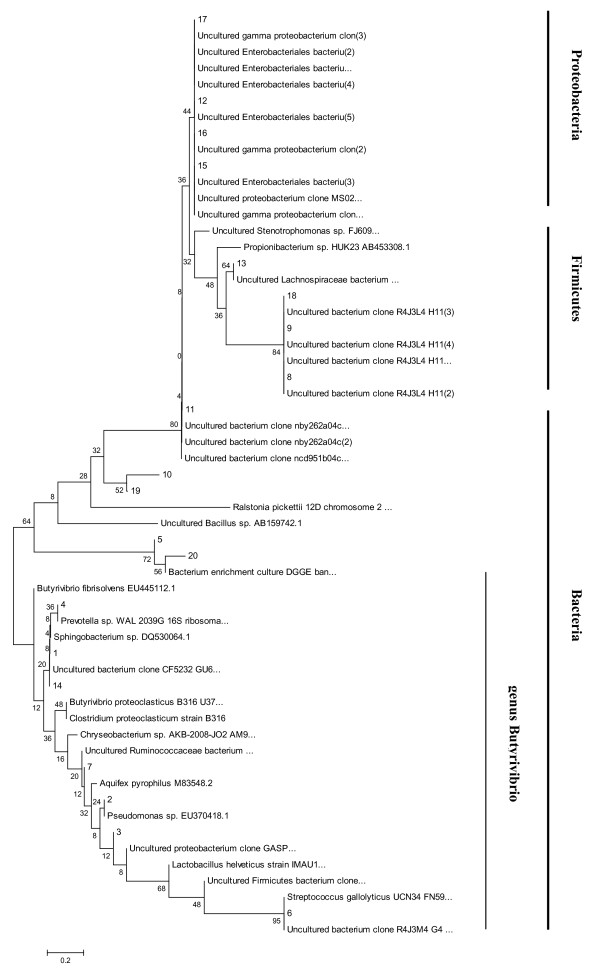
**Phylogenetic tree of bacterial 16S rRNA gene sequences retrieved from the t-VA enrichment cultures**.

Phylogenetic analysis of the predominant bacterial community, as visualized in the DGGE patterns of stabilized enrichment cultures, was accomplished after 16S rRNA gene cloning and sequence analysis (Figure 5). Phylogenetic analysis revealed that all of the sequenced bacterial clone bands in the t-VA treatment clustered into the *Proteobacteria, Firmicutes *and *Bacteria *phyla. Only seven bacterial sequences were related to *Butyrivibrio *spp, and the other nine mainly fell into *Proteobacteria *and *Firmicutes*. The four bacterial sequences that existed in both control and t-VA treatment were related to *Enterobacteriales bacteria *(*Proteobacteria *phylum), *Lachnospiraceae bacterium *(*Firmicate *phylum).

## Discussion

Griinari and Corl, et al. [5] (2000) showed that the close relationship between t-VA and CLA in milk fat is related to the formation of cis-9, trans-11 CLA from t-VA via Δ^9^-desaturase. This close relationship has also been observed over a wide range of t-VA concentrations. The focus has been on ruminal formation of t-VA rather than CLA. In practical terms, this means that the most feasible options to enhance milk fat CLA concentrations may be to feed supplements containing t-VA or with dietary management of rumen biohydrogenation to increase the formation of t-VA. However, in mixed ruminal bacteria, t-VA was further hydrogenated to SA [8] (Ward and Scott et al., 1964). Up to now, only a small subgroup within the *Butyrivibrio fibrisolvens *grouping carries out the reduction of 18:1 fatty acids, particularly t-VA to SA [9] (Wallace and Chaudhary et al., 2006). This subgroup is now known as *Butyrivibrio proteoclasticus *[10] (Moon and Pacheco et al., 2008), formerly *Clostridium proteoclasticum *[11] (Wallace and Chaudhary et al., 2006), which in turn is believed to be the same species as that isolated over three decades ago, named *Fusocillus *spp [12] (Kemp and White et al., 1975). In the above studies, oleic acid, linoleic acid or linolenic acid acted as the substrate to isolate ruminal hydrogenated t-VA bacteria according to the prior pathway unsaturated fatty acid in the rumen: metabolism of unsaturated fatty acids [2] (Jenkins and Wallace et al., 2008). However, Erin [13] (2002) concluded that biohydrogenation of oleic acid by mixed ruminal microbes involves the formation of several positional isomers of trans monoenes rather than only direct biohydrogenation to form SA as previously described. The objective of this study revisited the ruminal bacteria which participate in the biohydrogenation of t-VA. In this study, t-VA was the main substrate of unsaturated acid.

It was essential to use mixed microbes in the cultures instead of a single species. The identification of a specific microbial species that can perform all of the steps of biohydrogenation would be difficult. Many different pure bacterial strains are negligible or incomplete [14] (Harfoot and Hazelwood, 1988). Ruminal bacteria are symbiotic in that they exchange intermediates of biohydrogenation between populations. Therefore, ruminal bacteria are grouped according to which substrate is used during the process of biohydrogenation [7] (Kemp and Lander, 1984). Furthermore, single bacterial species are not recognized for having the ability to carry out biohydrogenation.

Margarida et al. showed the rates of metabolism of VA in the rumen tended to be the same and were lightly influenced by the other products of metabolism when ruminant microbes were incubated after 12 h in vivo [15] (Margarida and Lal et al., 2007). The bacterial densities in the tubes were the same after 12 h as after one day in this study (data no shown), which were consistent with the previous studies.

When purified t-VA was added to cultures of mixed ruminal microbes, the concentration of SA initially decreased and subsequently increased. Perhaps the concentrations of t-VA used were high enough to reveal quantitative patterns not only of metabolism, but also of toxicity, which is relevant to microbial ecology and nutrition [2] (Jenkins, 2008). At the initial period of culture, on the one hand, some kinds of ruminal bacteria do not adapt to the in vitro condition. On the other hand, ruminal bacteria inhibited by t-VA did not exist in the t-VA medium. This is the primary reason that biohydrogenation product of t-VA in the t-VA treatment was decreased (Figure 1). After numerous transfers, t-VA in the medium was converted into SA according to the enrichment of hydrogenated bacteria. The ratio of t-VA transformed to SA showed that enriched ruminal bacteria participated in biohydrogenation and had performed this transformation (Figure 2). However, the concentration of t-VA in the treatment group was slightly increased. It is possible that the ruminal inoculum contains amounts of unsaturated fatty acid as precursors of t-VA like cis-9-18:1 [14] (Harfoot and Hazlewood, 1988) (data was not provided). This effect was negligible in this study.

According to the changed bands density in the DGGE profiles, bacteria community changed during the successive transfers in the cultures. The highly enriched t-VA hydrogenated cultures were obtained by successive transfers. DGGE analysis of t-VA enrichment cultures and non-t-VA cultures showed different bacterial compositions. There were approximately 20 different bands in the t-VA enrichment compared to the control group, which perhaps was representative of ruminal biohydrogenation bacteria. Also, 4 main bands were present in the control group and t-VA treatment group which means that either considerable amounts of ruminal bacteria did not participate in the biohydrogenation of t-VA or they belonged to the symbiotic group.

The diversity of both cultures has the similar trend (Figure 4), which showed that the diversity of ruminal bacteria was not related to the composition of the medium with the exception of cultivation time. This phenomenon perhaps suggests that normal microbial growth and metabolism was affected by in vitro conditions such as pH value [16] (VanSoest, 1994).

Phylogenetic analysis based on 16S rRNA sequence analysis of ruminal bacteria indicated that the stearate producers clustered on a branch with *Butyrivibrio proteoclasticus *[10] (Moon and Pacheco et al., 2008), formerly *Clostridium proteoclasticum *[2,11] (Wallace and Chaudhary et al., 2006; Jenkins and Wallace et al., 2008). However, ruminal bacteria are symbiotic between populations, especially the group which hydrogenates t-VA. The different band was representative of a different group of bacteria (Figure 3). Analysis of the microbial composition of t-VA enrichment cultures showed that some of the bacteria, as detected by DGGE analysis, were most closely related to uncultured bacteria, and all belonged to *Firmicutes *and *Proteobacteria *phyla (Figure 5). Hence, these microorganisms might play an important role in the biohydrogenation. Seven predominant bands in the enrichment cultures showed affiliation with the *Butyrivibrio *genus. High correlation between 16S rRNA gene sequences of clones 1, 4, 14 indicated that they most likely represent the same species, clustering within the *Butyrivibrio *genus. Sequences of clones 2, 3, 6, 7 were more distant from *Butyrivibrio fibrisolvens*. The low 16S rRNA gene sequence correlation of these microorganisms with cultured species makes it difficult to speculate about their physiological capabilities. However, t-VA enrichment cultures gave insight into the role of bacteria from which 20 clones were derived (Figure 5).

## Conclusions

Our study demonstrates that different ruminal bacteria belonging to *Firmicutes *and *Proteobacteria *families, and especially *Butyrivibrio proteoclasticus*, participate in the biohydrogenated conversion of t-VA to SA. However, most of the ruminal bacterial participating in biohydrogenation are uncultured. Although isolation of pure cultures from syntrophic associations is difficult due to low growth rates and mutual dependence of partner bacteria, their isolation in pure culture would help to confirm their role in t-VA conversion in future. Doing so would give more insight into the pathway of biohydrogenation. Moreover, it may be an important step in the management of ruminal biohydrogenation bacterium to control the production of t-VA, which has metabolic and physiological benefits.

## Methods

### Culture media composition and cultivation

Mixed ruminal microbes were obtained from the rumen of 6 cows with surgically prepared rumen fistulas [17] (Erin, 2010). Ruminant bacterium were prepared using sedimentation procedure, as previously described by Estelle et al. [18] (2006), and were inoculated into M2 medium [19] (Hobson, 1969), which contained 50 μg t-VA ml^-1 ^(Sigma, America) [20] (Nest and Kevin et al., 2010). t-VA was prepared as a separate solution, sonicated for 4 min in water and added to the medium before autoclaving [15] (Margarida and Lal et al., 2007). Enrichment series were obtained by successive transfers of active cultures (10%) into new medium containing 50 μg t-VA ml^-1 ^as treatment groups (Trt) and culture without t-VA as control groups (CK) at 12-h intervals [15] (Margarida and Lal et al., 2007). Cultures were sampled at 12-h intervals to determine fatty acid (FA) concentration, and DNA was extracted for DGGE analysis. Incubations were performed in triplicate. All transfers and incubations were carried out in pure CO_2 _and at 39°C in anaerobic tubes.

### Fatty acid analysis

Culture samples were converted to methyl esters in sodium methoxide-methanolic HCl as described by Kramer and Fellner et al. [21] (1997). Analysis of culture medium fatty acids was done on a high performance gas chromatograph (HP5890A GC, CA) equipped with a flame-ionization detector and a 30 m × 0.25 mm (0.2 μm film) Supelco 2380 fused-silica capillary column. The injector and detector temperatures were held at 250 and 260°C, respectively. The carrier gas was He (20 cm s^-1^) with an inlet pressure of 104 kPa. The column temperature was programmed for 140°C for 3 min, then increased to 220°C at 2°C min^-1^, and held at 220°C for 2 min. Peaks were quantified by comparison with an internal standard (17:0), which was added prior to methylation.

### Evaluation of data

Data were analyzed by the PROC general linear model (GLM) at each time point. All results are expressed by their least square means (LSMEANS). The significance of the difference (*P *< 0.05) was assessed using SAS (SAS Institute, Inc., Cary, NC)

### DNA extraction

Total DNA of ruminal microbe was extracted using the RBB+C method [9] (Zhongtang and Mark et al., 2004). Cell lysis is achieved by bead beating in the presence of 4% (w/v) sodium dodecyl sulfate (SDS), 500 mM NaCl, and 50 mM EDTA. After bead beating, most of the impurities and the SDS are removed by precipitation with ammonium acetate, and then the nucleic acids are recovered by precipitation with isopropanol. Genomic DNA can then be purified via sequential digestions with RNase and proteinase K, followed by the use of QIAamp columns.

### PCR and DGGE analysis

The V3 region of the *rrs *gene was amplified by PCR using primers 338f and 533r [22] (Huse and Dethlefsen et al., 2008). The 338f primer has a 40-base GC clamp [23] (Mako and Eiichi et al., 2002) attached to its 5'end. All PCR amplifications were performed using a PTC-100^® ^Peltier Thermal Cycler (MJ Research, USA) in 50 μL volumes containing 1 × PCR buffer (20 mM Tris-HCl, pH 8.4, and 50 mM KCl), 200 μM dNTP, 500 nM each primer, 1.75 mM MgCl2, 670 ng μL^-1 ^bovine serum albumin (BSA), and 1.25 U Platinum^® ^Taq DNA polymerase (Invitrogen, USA). After an initial denaturation at 94°C for 4 min, 10 cycles of touchdown PCR were performed (94°C for 30 s, 61°C for 30 s, with a 0.5°C per cycle decrement, and 72°C for 1 min), followed by 25 cycles of PCR (94°C for 30 s, 56°C for 30 s, and 72°C for 1 min), and a final extension step at 72°C for 7 min. 15 μL aliquots were resolved in a 7.5% polyacrylamide gel (37.5:1) containing a 40%-60% gradient of denaturants [100% denaturants consisting of 40% (v/v) formamide and 7 M urea]. The DGGE gel was run at 60°C and 82 V for 15 h using a DCode™ Universal Mutation Detection System (Bio-Rad Laboratories, USA). The DGGE gel was then stained with GelStar (Cambrex, USA), and the gel images were captured using a FluorChem Imager (Alpha Innotech).

### DGGE analysis of PCR amplicons

Gels were analyzed using the Quantity One software package, version 4.62 (Bio-Rad Laboratories, USA). After normalization, bands were defined for each sample by using band detection based on parameters and the standard lanes were identified, and determine the values of the experimental bands using those standards. Peak heights in the densitometric curves were used to determine the diversity indices based on the Shannon-Weiner diversity index, calculated as *H *= -Σ[*P*_i_ln(*P*_j_)], where *H *is the diversity index and *P*_i _is the importance probability of the bands in a lane (*P*_j _= n_j_/n where n_i _is the height of an individual peak and n is the sum of all peak heights in the densitometric curves).

### Sequencing of DGGE fragments

Predominant bands in the DGGE gel of 16S rRNA fragments were excised with a razor blade. The DNA was retrieved from the acrylamide block with Centriluter (Amicon) at 150 V for 3 h. The DNA was reamplified with 338f and 533r. PCR products were cloned into *Escherichia coli *JM109 (TaKaRa, China) by using the pMD18-T Easy vector system (TaKaRa, China). The plasmid DNA was extracted using the Plasmid Purification kit (TaKaRa, China), and was screened by amplified ribosomal DNA restriction analysis (ARDRA), using the restriction enzymes MspI, Cfol, and AluI (TaKaRa, China). The restriction fragments were analyzed by electrophoresis in agarose gel and visualized with ethidium bromide. Plasmids of selected transformants, with different ARDRA patterns and corresponding to predominant bands in the DGGE community fingerprint, were subjected to DNA sequence analysis. The consensus sequences obtained were checked for potential chimera artifacts by the CHECK CHIMERA program of Ribosomal Database Project II (RDP release 8.1, http://rdp.cme.msu.edu/) [24] (Cole, 2003). 16S rRNA sequence data have been deposited to the NCBI database (http://www.ncbi.nlm.nih.gov/ Genbank) with accession numbers from JQ284436 to JQ284447.

### Phylogenetic placement

Similarity searches for the 16S rRNA gene sequences were performed using the NCBI BLAST search program within the GenBank database [25] (Altschul and Gish et al., 1990). Alignment of the 16S rRNA sequences was performed by using the MEGA 4 program. The resulting alignments were manually checked and corrected when necessary, and unambiguously aligned nucleotide positions were used for the construction of a 16S r-RNA gene-based phylogenetic tree by using the neighbor-joining method [26] (Saitou and Nei, 1987).

## Competing interests

The authors declare that they have no competing interests.

## Authors' contributions

The contribution of each author to the present paper was as follows: DLi and JW designed the study and analysed the data; DB collected the data; DL wrote the draft of the manuscript. All authors read and approved the final manuscript.
